# Molecular identification of methane monooxygenase and quantitative analysis of methanotrophic endosymbionts under laboratory maintenance in *Bathymodiolus platifrons* from the South China Sea

**DOI:** 10.7717/peerj.3565

**Published:** 2017-08-07

**Authors:** Yan Sun, Minxiao Wang, Leilei Li, Li Zhou, Xiaocheng Wang, Ping Zheng, Haiyan Yu, Chaolun Li, Song Sun

**Affiliations:** 1 Key Laboratory of Marine Ecology and Environmental Sciences, Institute of Oceanology, Chinese Academy of Sciences, Qingdao, China; 2 University of Chinese Academy of Sciences, Beijing, China; 3 State Key Laboratory of Microbial Technology, Shandong University, Jinan, China; 4 Laboratory for Marine Ecology and Environmental Science, Qingdao National Laboratory for Marine Science and Technology, Qingdao, China; 5 Jiaozhou Bay Marine Ecosystem Research Station, Chinese Ecosystem Research Network, Qingdao, China

**Keywords:** Bathymodiolus platifrons, Methane monooxygenase, Methane, South China Sea, Symbiont abundance

## Abstract

Deep-sea mussels of the genus *Bathymodiolus* are numerically dominant macrofauna in many cold seep and hydrothermal vent ecosystems worldwide, and they depend on organic carbon produced by symbionts present in the epithelial cells of the gills. Although *Bathymodiolus platifrons* represents typical methanotrophic endosymbiosis, our understanding of molecular mechanisms of methane oxidization and carbon fixation is still in its infancy. Moreover, the laboratory maintenance of *B. platifrons* and the symbiont abundance dynamics during maintenance has not been reported. In the present study, we report the first systematic identification and phylogenetic analysis of three subunits of methane monooxygenase (pmoA, pmoB, and pmoC) obtained from the endosymbiotic bacteria found in *B. platifrons*. The coding sequences (CDS) of the three genes in the *B. platifrons* endosymbiont were 750, 1,245, and 753 bp, encoding 249, 414, and 250 amino acids, respectively. Sequence alignment and phylogenetic analysis revealed that the symbiont of *B. platifrons* belongs to the type I methanotrophs. In order to clarify the impact of environmental methane on symbiont abundance, a 34-day laboratory maintenance experiment was conducted in which *B. platifrons* individuals were acclimatized to methane-present and methane-absent environments. Symbiont abundance was evaluated by calculating the relative DNA content of the methane monooxygenase gene using quantitative real-time PCR. We found that symbiont quantity immediately decreased from its initial level, then continued to gradually decline during maintenance. At 24 and 34 days of maintenance, symbiont abundance in the methane-absent environment had significantly decreased compared to that in the methane-present environment, indicating that the maintenance of symbionts relies on a continuous supply of methane. Our electron microscopy results validated the qPCR analysis. This study enriches our knowledge of the molecular basis and the dynamic changes of the methanotrophic endosymbiosis in* B. platifrons*, and provides a feasible model biosystem for further investigation of methane oxidization, the carbon fixation process, and environmental adaptations of deep-sea mussels.

## Introduction

Deep-sea mussels of the genus *Bathymodiolus* (Bivalvia: Mytilidae) are one of the most successful macrofauna in terms of biomass in many cold seep and hydrothermal vent ecosystems worldwide (Sibuet & Olu, 1998; Van Dover, 2000). In such environments, with a total absence of sunlight, symbiosis with chemosynthetic bacteria is a key physiological adaptation achievement. These are located in specialized gill epithelium called bacteriocytes (Fisher, 1990), and fix inorganic carbon from the environment to provide a source of nutrition to the host mussels (Duperron et al., 2009). The most common endosymbionts in deep sea macrofauna are thiotrophic and methanotrophic bacteria, with the latter being the most often reported in mytilid mussels (Petersen & Dubilier, 2009). Many *Bathymodiolus* species possess two or more distinct endosymbionts coexisting in their gill bacteriocytes allowing for a flexible process of nutrition acquisition. Therefore, deep-sea *Bathymodiolus* spp. are regarded as good models to study host-symbiont relationships.

The species *B. platifrons* is found at a hydrothermal vent in the Okinawa Trough and cold seeps in Sagami Bay (Japan) and the South China Sea (Fujiwara et al., 2000; Barry et al., 2002; Feng et al., 2015). High concentrations of methane are recorded in these habitats, and a single species of methane-oxidizing bacteria is the only type of endosymbiont that has been found in its gill using transmission electron microscopy and 16S ribosomal RNA gene sequencing (Fujiwara et al., 2000; Barry et al., 2002). The geographic distribution of *B. platifrons* is strongly influenced by the local methane concentration (Gamo, 1995; Ishibashi et al., 1995; Fujiwara et al., 2000), and stable isotope ratios of carbon have revealed that methane is its major carbon source (Feng et al., 2015). Therefore, methane oxidization is critical for the survival of *B. platifrons* in extreme deep-sea environments. However, limited information is available regarding the underlying molecular mechanisms.

To gain insight into the genetic basis of the methane-oxidizing and carbon fixation processes in *B. platifrons*, the development of a long-term maintainable system under laboratory environment is essential. Unlike many other deep-sea invertebrates, some relatively shallow *Bathymodiolus* species, such as *B. azoricus* from Menez Gwen at 850 m depth and *B. childressi* from the Louisiana Slope at 580 ∼ 700 m depth, can survive and maintain their endosymbionts active at atmospheric pressure for a long time under laboratory conditions (Martins et al., 2016; Kochevar et al., 1992; Arellano & Young, 2009). Nevertheless, differences between the deep-sea *in situ* environment and the laboratory environment, for example the substrate concentration, may lead to abundance changes of the endosymbiont during maintenance (Riou et al., 2008; Guezi et al., 2014; Fujinoki et al., 2012). In addition, acute environmental changes during collection can induce high cell stress and disturb the host-symbiont redox homeostasis, leading to the breakdown of symbiosis (Mone, Monnin & Kremer, 2014). Changes in symbiont abundance during maintenance are important for understanding the cross-talk of symbiont and host, the interaction between environment and symbiont, and to gain a stable endosymbiosis for future analysis. However, as a typical model for methanotrophic endosymbiosis, the maintenance of *B. platifrons* and the symbiont abundance dynamics during collection and under laboratory environments have not been studied.

FISH (fluorescent *in situ* hybridization) is a popular technique for detecting the presence and evaluating the relative abundance of endosymbionts, and has been widely used in studies on bacterial symbiosis in various invertebrates inhabiting deep-sea hydrothermal vents and cold seeps (Blazejak et al., 2005; Suzuki et al., 2005; Tokuda et al., 2008). Although FISH is an indispensable tool for visualizing the distribution of symbionts, the time-consuming procedure makes it difficult to detect large-scale samples simultaneously. Recent studies have used real-time qPCR to detect symbiont ratios in *in situ* or in laboratory environments for *Bathymodiolus* species (Boutet et al., 2011; Guezi et al., 2014; Detree et al., 2016). The qPCR method is a fast and easy approach for detecting changes in symbiont gene copy numbers of relatively large-scale samples.

Methane monooxygenase is a typical target gene for quantification analyses of methanotrophic endosymbionts in *Bathymodiolus* mussels, because it is a unique and key enzyme in methanotrophic bacteria, catalyzing the oxidation of methane to methanol, which is the first step of methane oxidation. Methane monooxygenase is comprised of three subunits, pmoA, pmoB and pmoC, which are encoded by the operon *pmoCAB* (Ward et al., 2004). Many *pmoA* genes have been identified in the symbionts of deep-sea mussel species, including *B. azoricus*, *B. heckerae*, *B. brooksi* and *B. childressi* (Duperron et al., 2007; Raggi et al., 2013; Spiridonova et al., 2006). The molecular identification of *pmoA* in deep-sea mussels not only provides a specific target gene for FISH analysis, but also lends support to phylogenetic analyses constructed from 16S rDNA sequences. The *pmoB* and *pmoC* genes have not yet been reported in deep-sea mussels, although they also possess metal centers/active sites critical to the function of methane monooxygenase (Culpepper & Rosenzweig, 2012; Lawton et al., 2014).

In the present study, we focused on the key methane oxidation genes in a *B. platifrons* symbiont and report the first systematic identification and phylogenies of three subunits of methane monooxygenase (pmoA, pmoB, and pmoC). Furthermore, we describe the first sustained laboratory maintenance experiment of *B. platifrons* to analyze the symbiont dynamics during maintenance and the impact of environmental methane on symbiont abundance, using real-time qPCR validated with electron microscopy. Our results help for understand the molecular basis and the dynamic changes under CH_4_ of methanotrophic endosymbiosis in *B. platifrons*.

## Materials and Methods

### Ethics statement

All mussels handling was conducted in accordance with the guidelines and regulations established by the Institute of Oceanology, the Chinese Academy of Sciences, and the China Government Principles for the Utilization and Care of Animals Used in Testing, Research, and Training (State science and technology commission of the People’s Republic of China for No. 2, October 31, 1988: http://www.gov.cn/gongbao/content/2011/content_1860757.htm).

### Database mining, gene identification, and sequence analysis

Three subunits of methane monooxygenase, abbreviated as *pmoA*, *pmoB* and *pmoC*, were identified from draft genome data of the *B. platifrons* methanotrophic endosymbiont (L Li, 2017, unpublished data) by TBLASTN, using the homologous pmoA, pmoB and pmoC of *Methylococcus capsulatus* (Bath) as query sequences with an *E*-value cutoff of 1*e* − 8. The methanotrophic endosymbiont draft genome data were obtained from metagenomic sequencing and assembly of gill tissue of *B. platifrons* from a cold seep in the South China Sea (22°06′57.144″N, 119°17′6.580″E). The methane monooxygenase amino acid sequences were predicted by DNAstar (version 4.05) and further verified by BLASTP against the NCBI non-redundant (nr) protein database (https://blast.ncbi.nlm.nih.gov/Blast.cgi).

### Phylogenetic analysis

Homologous protein sequences and their corresponding coding sequences of methane monooxygenase subunits from type I, type II, and type X methanotrophs were obtained through online BLASTP search through database at NCBI using *B. platifrons* symbiont methane monooxygenase proteins as queries. Alignments of proteins were conducted in MAFFT v7 (Katoh & Standley, 2013). Coding sequences were aligned according to the protein alignments. ModelGenerator (Keane et al., 2006) was used to choose an appropriate model of sequence evolution for the alignment. Bayesian method was used to construct protein and coding sequence (CDS) phylogenetic trees as implemented in MrBayes 3.2 (Huelsenbeck et al., 2001; Ronquist et al., 2012).

### Mussel collection and CH_4_ treatment experiment

About 100 *B. platifrons* individuals were collected from a methane seep population at 1,113 m on the continental slope of the South China Sea (22°06′57.144″N, 119°17′6.580″E), using the remotely operated vehicle (ROV) ‘*Faxian*’. The gill tissues of five mussels were immediately dissected and frozen in liquid nitrogen. These freshly collected (FC) gill tissues were stored at −80 °C and used for control comparisons for the CH_4_ treatment experiment.

The remaining individuals were transferred to filtered seawater in an aquarium at atmospheric pressure. The temperature of the seawater was regulated to 4 °C, comparable to the temperature where they were collected. After acclimation for one day, they were divided into two groups of 40 individuals apiece and maintained in methane-supplied (CH_4_ +) or methane-absent (CH_4_ −) environments. Group 1 (CH_4_ +) was perfused with methane gas for 30 min twice every day, while group 2 (CH_4_ −) received no additional methane. The gills from five individuals in each group were collected on days 0, 7, 14, 24, and 34 of the experiment.

In order to detect the solution efficiency and escape rate of methane in the aquarium, the dissolved methane concentration was measured by gas chromatography. Fifteen milliliters of the seawater were subsampled in a time series from 30 min to 300 min after methane was perfused into the seawater without mussels. Water subsampling and methane concentration measurements were performed in triplicate at every time-point.

### DNA extraction

Genomic DNA from the gills was extracted using an E.Z.N.A.^®^ Mollusc DNA Kit (Omega Bio-Tek, Norcross, GA, USA) with one minor change, where an additional digestion step was carried out after the initial digestion of the gill tissues with lysozyme (20 µl, 50 mg/ml) at 30 °C for 10 min. This ensured complete release of the bacterial endosymbionts’ DNA. DNA quality and quantity was assessed using 1% agarose gel electrophoresis and a Nanodrop 1000 spectrophotometer (Thermo Scientific, Wilmington, DE, USA).

### Detection of symbiont quantity by real-time PCR

Real-time PCR procedures were performed using an Eppendorf Realplex^4^ thermocycler (Eppendorf, Hamburg, Germany) using FastStart Essential DNA Green Master (Roche Diagnostics, Mannheim, Germany), 4 µM of each primer, and 20 ng DNA in a final volume of 20 µl. The PCR program was as follows: 2 min at 95 °C, followed by 40 cycles of 15 s at 95 °C, 15 s at 59 °C, and 20 s at 72 °C. Additional melting curve analysis was performed at the end of each PCR to test the specificity of the amplification reaction. qPCR was performed in triplicate for each gene and mussel specimen.

**Table 1:** Primers used in real-time PCR amplification of bacteria methane monooxygenase and host reference gene.

Gene name	Primer sequence (5′ − 3′)	Tm (°C)	Amplicon length (bp)	Amplification efficiency
*pmoA*	F: TGGACAGATTGGAAAGATAGACG	59	111	0.96
	R: GAAAGGCAGACGGTAACGG			
*pmoB*	F: CCTATTACAACTCCATTACAAGCG	59	117	0.96
	R: AGCACGACCAGGAACACGA			
*pmoC*	F: GCAGGTTGGATGTTGTATTTAGTG	59	99	0.94
	R: GAACTCAGGTGCGAATGAATCTA			
*β*-*actin* (host gene)	F: GACGAAGCCCAGGTAAAACG	59	198	1.00
	R: CTTAGTCATCATTTCTCTGTTGCCT			
*Ribosomal protein L15* (host gene)	F: GGGTAGCCCAGGACTCTTCA	59	130	0.93
	R: CCACGCATTTCTCTGTGTTTG			

Primer information for the symbiont and host genes is listed in Table 1. The PCR products were sequenced to confirm the primer specificity by Shanghai Sangon Biotechnology Co. Ltd. (Shanghai, China). Two host genes, *β-actin* (ACTB) and *ribosomal protein L15* (RPL15), were used as internal control genes, and the relative symbiont quantity was normalized by the geometric averaging of the two host genes (Vandesompele et al., 2002). The relative quantity of the symbiont was estimated using the 2^−ΔΔCt^ method (Livak & Schmittgen, 2001), in which ΔΔCt = (Ct_symbiont−gene_ − Ct_host−gene_)_Laboratory−maintained_ − (Ct_symbiont−gene_ − Ct_host−gene_)_Freshly−collected_. Following the formula, the relative symbiont quantities were normalized as fold differences relative to freshly collected mussels. *T*-tests was conducted between symbiont abundances of the CH_4_ + and CH_4_ − groups, and differences were considered statistically significant if *p* < 0.05.

### Validation of symbiont abundance by electron microscopy

Several gill filaments at the mid portion in the external demibranchs of the freshly collected and maintained (at the end of 34 days) mussels were dissected and fixed overnight in 2.5% glutaraldehyde/2% paraformaldehyde (Solarbio, Beijing, China) at 4 °C. For scanning electron microscopy (SEM) testing, samples were then washed three times in 0.1 M phosphate buffer (pH 7.4), dehydrated in a graded ethanol series (50, 70, 90, 95 and 100% for 10 min each) and subjected to critical point drying in a Critical Point Dryer (Leica, EM CPD300, Germany). The dried specimens were transferred to an adhesive tape on a specimen stub, then coated with gold by an ION Sputtering Device (108; Cressington, Watford, UK), and observed with a field emission SEM (Quanta FEG 250; FEI, Hillsboro, OR, USA) operated at 10 kV accelerating voltage and 3.0 spot size.

For transmission electron microscopy (TEM) testing, samples were washed three times in 0.1 M phosphate buffer (pH 7.4) after the first fixation. Post-fixation was in cold 1% aqueous osmium tetroxide for 1 h, then samples were washed three times in 0.1 M phosphate buffer (pH 7.4). Samples were dehydrated in a graded ethanol series (50, 70, 90, 95, and 100% for 10 min each) and 100% acetone three times (10 min each), and then permeated in a gradient of epon 812 and acetone (1:3, 1:1, 3:1, pure epon 812, 60 min each;), and embedded in epon 812 (60 °C, 36 h). Ultrathin sections were sliced with glass knives on an ultra microtome (Powertome-XL; RMC/Boeckeler, Tuscon, AZ, USA). Sections were stained with uranyl acetate and lead citrate and examined under a TEM (Tecnai F20; FEI, Hillsboro, OR, USA).

TEM results were employed to validate the symbiont abundance in the gill bacteriocytes according to the method described by Laurich, Batstone & Dufour (2015) using ImageJ (Rasband, 1997). First, total cell area of a bacteriocyte (Bc_A_) was measured after manually outlining the area enclosed by the bacteriocyte cell membrane. Subsequently, the symbionts were manually delimitated and their total area (S_A_) was measured. Then, the symbiont abundance (S_Abd_) was calculated as the ratio of the area occupied by bacterial symbionts to the total cell area of the bacteriocyte (*S*_Abd_ = *S*_A_∕Bc_A_). We calculated the symbiont abundance of five bacteriocytes at the median region on the gill filaments in FC mussel and CH_4_ + (34 d) mussel, and three bacteriocytes in CH_4_ − (34 d) mussel.

## Results

### Identification of methane monooxygenase subunits from *B. platifrons* symbionts

Three subunits of methane monooxygenase, *pmoA*, *pmoB*, and *pmoC*, were identified from draft genome data of *B. platifrons* endosymbionts. They shared high sequence identity with pmoA, pmoB and pmoC of *Methylococcus capsulatus* (Bath). The CDS of *pmoA*, *pmoB*, and *pmoC* of *B. platifrons* endosymbionts were 750, 1,245, and 753 bp, encoding 249, 414, and 250 amino acids, respectively (Figs. S1–S3). The CDS and deduced amino acid sequences of the three methane monooxygenase subunits were deposited in GenBank under accession numbers KY393122, KY393123, and KY393124.

**Figure 1:**
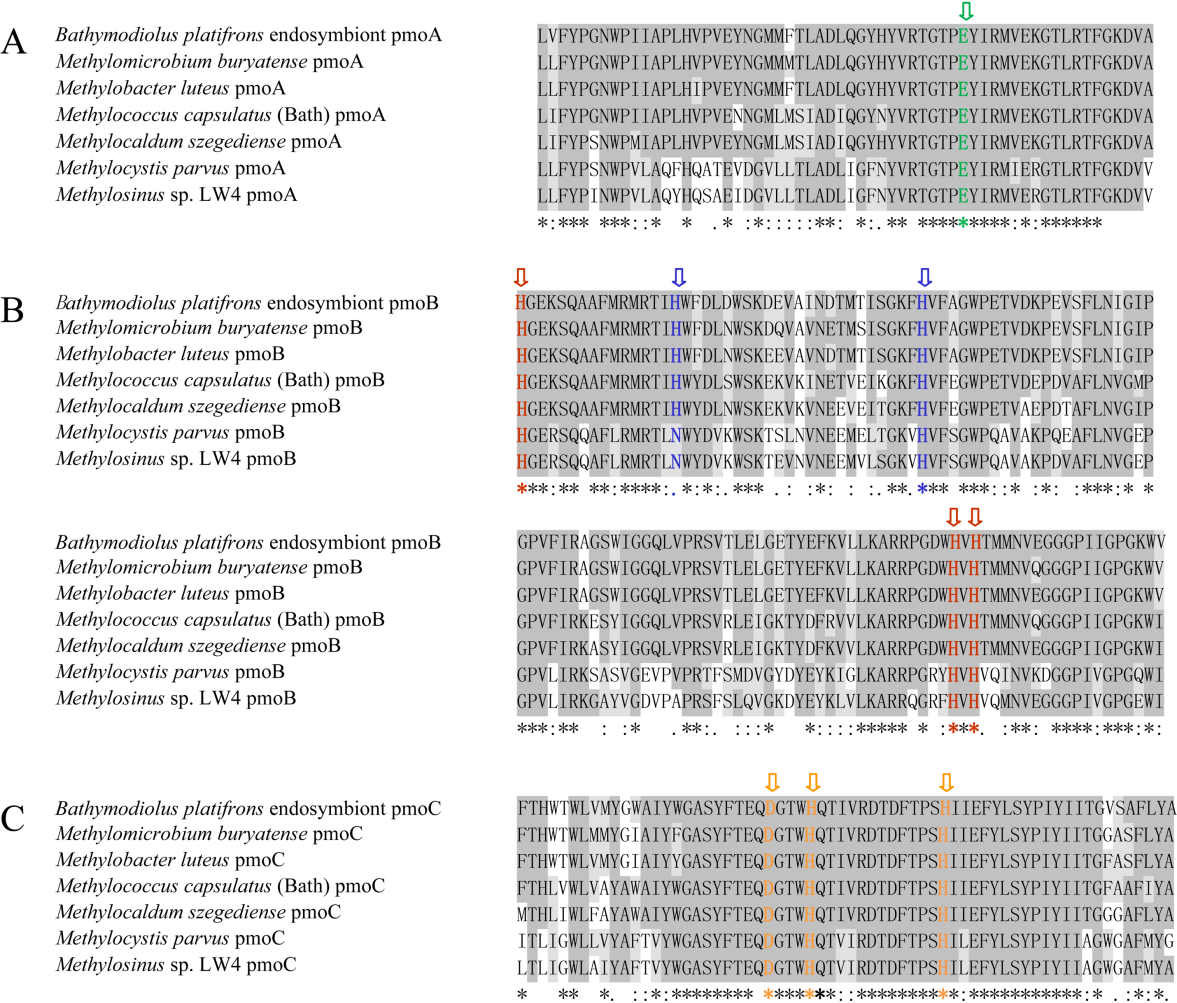
Amino acid sequence alignment of pmoA (A), pmoB (B) and pmoC (C) in *B. platifrons* endosymbiont compared to representative sequence from other methanotrophic bacteria. *Methylomicrobium buryatense* and *Methylobacter luteus* belong to the type I methanotrophs; *Methylocystis parvus* and *Methylosinus sp.* LW4 belong to the type II methanotrophs; and *Methylococcus capsulates* (Bath) and *Methylocaldum szegediense* belong to the type X methanotrophs. The critical amino acid residues of the three metal centers are marked with different colors. The dicopper and monocopper site metal centers are located in pmoB, and the amino acids ligands are shown in red and blue, respectively. The amino acids ligands of the zinc site are shown in green and orange in pmoA and pmoC.

Alignment of the amino acid sequences was performed for the three methane monooxygenase subunits of the *B. platifrons* endosymbiont and other methanotrophs, including type I (*Methylomicrobium buryatense*, *Methylobacter luteus*), type II (*Methylocystis parvus*, *Methylosinus sp.* LW4), type X (*M. capsulatus*, *Methylocaldum szegediense*) methanotrophs. Accession numbers are listed in Table S1. According to the results, the *B. platifrons* symbiont pmoA, pmoB, and pmoC shared high sequence similarity with methanotrophic bacterial orthologous proteins (Fig. 1), and the critical amino acid residues of three metal centers were conserved with those of other methanotrophs (Fig. 1).

### Similarity and phylogenetic analysis

PmoA, pmoB, and pmoC proteins from methanotrophic bacteria under three phylogenetic groups, type I, type II, and type X, were used for similarity and phylogenetic analysis. Similarity analysis was carried out based on amino acids sequences. The result (Table S1) indicated that *B. platifrons* symbiont pmoA, pmoB, and pmoC shared higher similarity to proteins in their corresponding subunits compared to other subunits. For instance, the similarity between *B. platifrons* symbiont pmoA and other pmoA orthologs ranged between 77.2% and 93.7%, and its similarity to pmoB and pmoC proteins ranged from 13.9–24.4% and 43.8-46.8%, respectively. Furthermore, the three subunit proteins of the *B. platifrons* symbiont shared higher sequence similarity to proteins in the type I methanotrophic subgroup.

**Figure 2:**
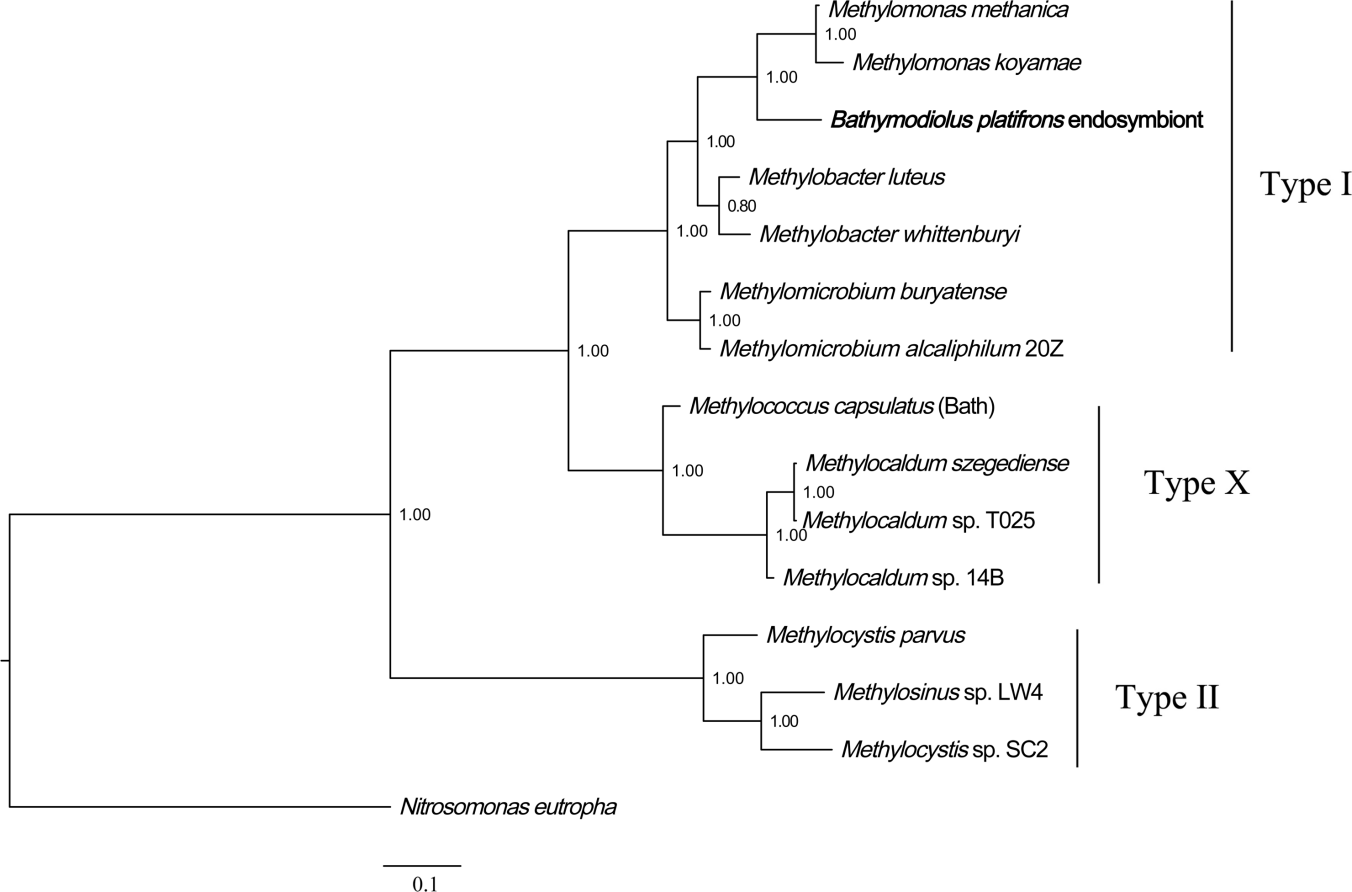
Phylogenetic reconstruction of methane monooxygenase based on Bayesian analysis of protein sequences. The 3 methane monooxygenase subunits pmoA, pmoB, and pmoC were concatenated before phylogenetic analysis. The posterior probability is indicated at the tree nodes. Methane monooxygenase of the *Bathymodious platifrons* symbiont is marked in bold. Accession numbers of proteins are given in Table S1. The ammonia monooxygenase of *Nitrosomonas eutropha* is used as an outgroup for the rooted tree. Accession numbers of *N. eutropha* ammonia monooxygenase subunits are: amoA, SFU84057; amoB, SFU84047; amoC, SFU84143. Bar: 0.1 substitutions per amino acid site.

Phylogenetic analysis of methane monooxygenase was performed based on Bayesian analysis of both amino acids and coding sequences using MrBayes 3.2 (Huelsenbeck et al., 2001; Ronquist et al., 2012). The three methane monooxygenase subunits were concatenated before phylogenetic analysis. For the amino acids tree, ModelGenerator indicated that the best-fit model for the combined methane monooxygenase proteins was LG substitution model (Le & Gascuel, 2008) and Gamma distributed (G). The ammonia monooxygenase of *Nitrosomonas eutropha* was used as an outgroup for the rooted tree. The result (Fig. 2) showed that *B. platifrons* symbiont methane monooxygenase was first clustered with the type I methanotrophic subgroup, and then clustered with type X and type II subgroups. The Bayesian phylogenetic tree of methane monooxygenase coding sequences was constructed based on the TVM substitution model (Posada, 2003) and Gamma distributed (G). The conformation of the CDS tree (Fig. S4) was in concordance with that of the protein tree. The branch length of the CDS tree was longer compared with the protein tree, indicating higher sequence variations in the coding sequences.

Bayesian phylogenetic analysis was also conducted based on the protein and coding sequences of three methane monooxygenase subunits, respectively. The results (Figs. S5 and S6) showed that the methane monooxygenase subunits clustered into three well-defined clades, corresponding to pmoA, pmoB and pmoC. In each clade, *B. platifrons* symbiont proteins were first clustered with homologs from the type I methanotrophic subgroup. The *B. platifrons* symbiont proteins were then clustered with homologs from type X and type II methanotrophic subgroups.

### Relative symbiont quantity of *B. platifrons* under CH_4_ influence

**Figure 3:**
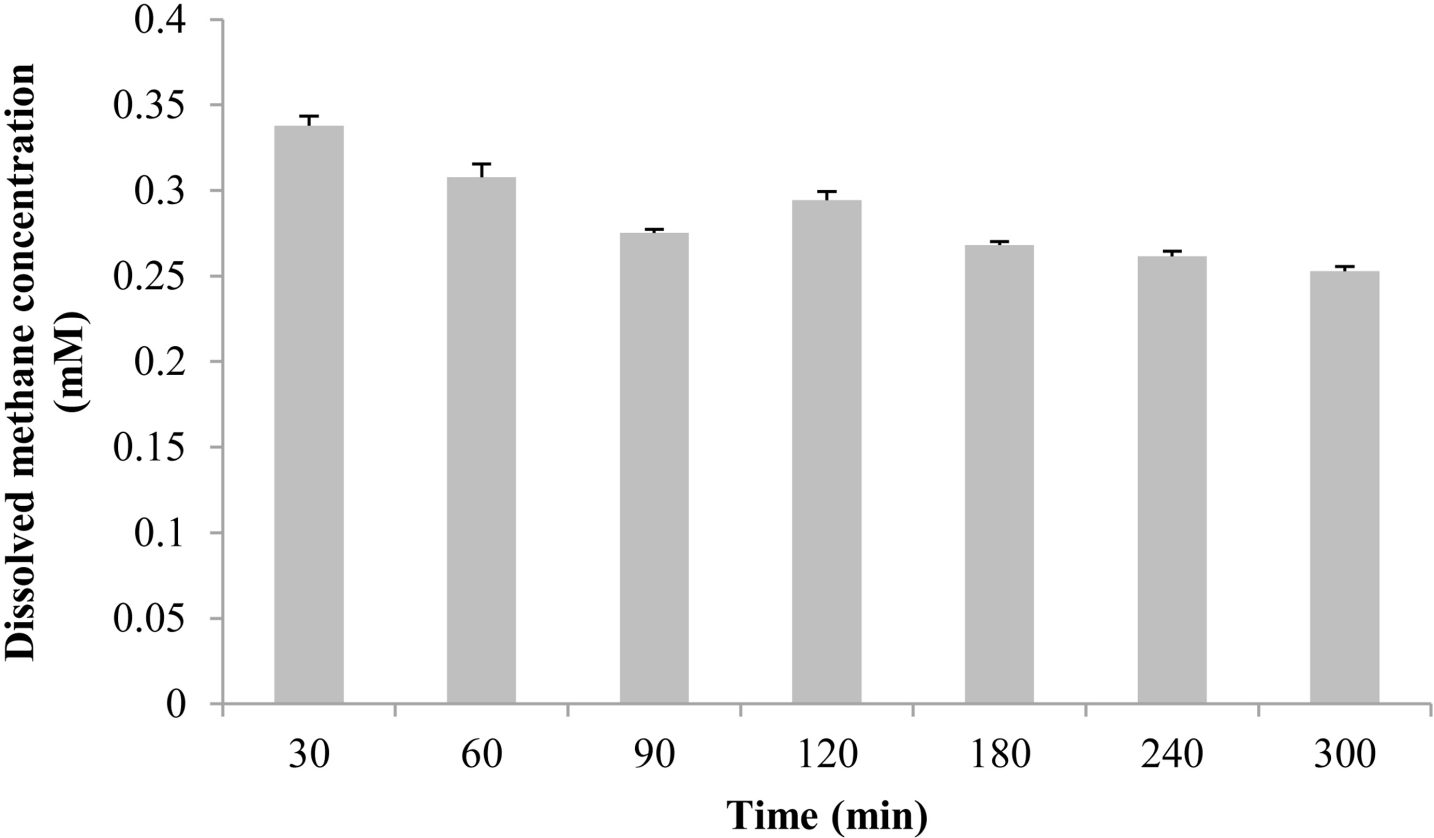
The dissolved methane concentration of seawater in an aquarium without mussels was measured by gas chromatography. The horizontal coordinates indicate the subsample time.

The measured methane concentration is shown in Fig. 3. After methane was perfused for 30 min, its concentration in the seawater was 0.338 ± 0.006 mM. Subsequently, the methane concentration slowly decreased, and was reduced to 0.253 ± 0.003 mM at 300 min.

**Figure 4:**
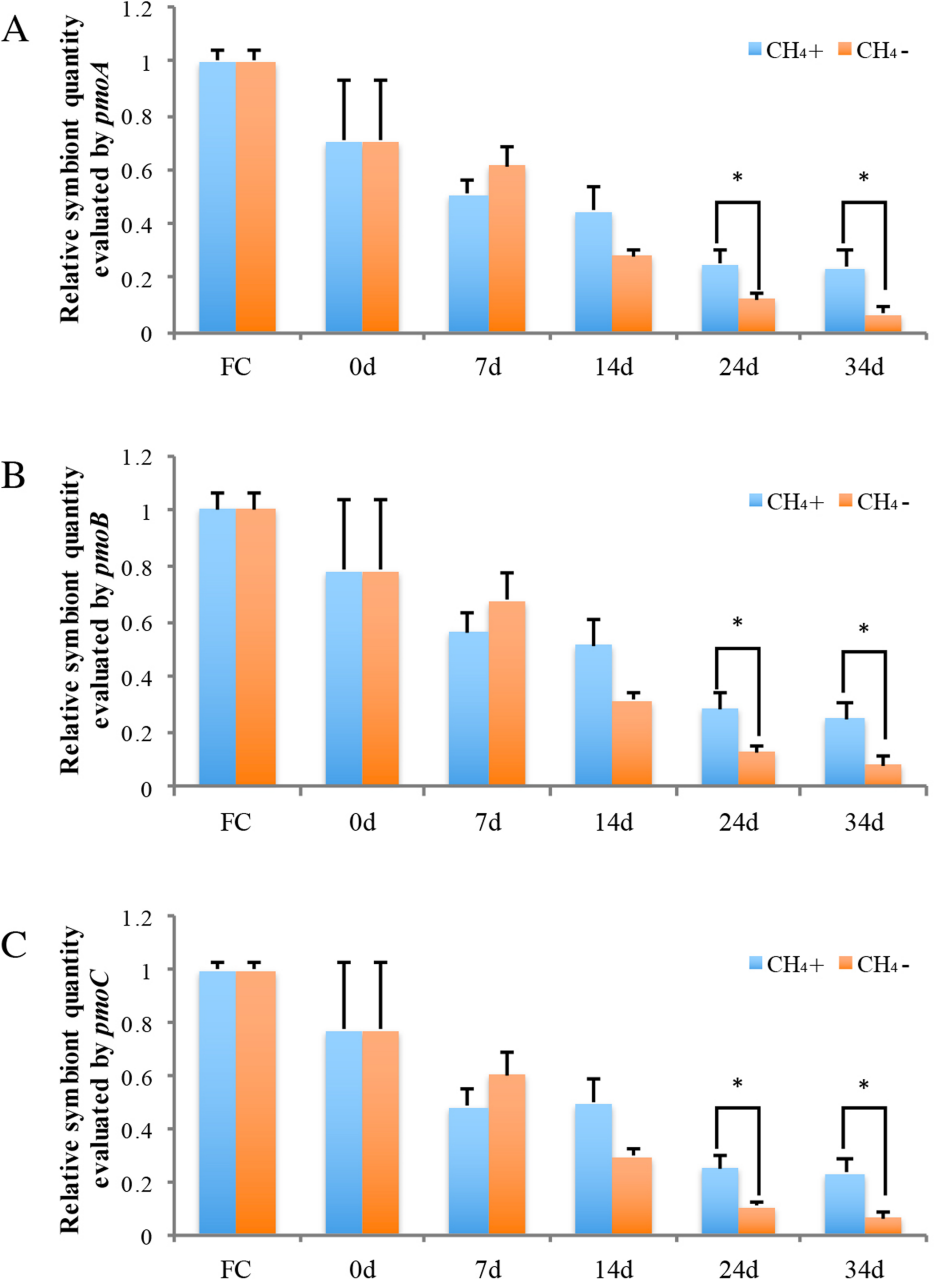
Relative symbiotic bacterial abanduance of symbiont genes *pmoA* (A), *pmoB,* (B) and *pmoC* (C) were evaluated by comparing their DNA content with host *β-actin* (ACTB) and *ribosomal protein L15* (RPL15) genes. “FC” indicates freshly collected mussels. The CH_4_ maintenance experiment was carried out after a one-day acclimation in the aquarium at atmospheric pressure. Quantitative analyses were made at 0 d, 7 d, 14 d, 24 d, and 34 d during maintenance. Relative symbiont quantities are shown as fold differences compared to that of FC. Group CH_4_ + and CH_4_ − indicate mussels maintained in methane-supplied and methane-absent environments, respectively. “*” indicates significant differences (*p* < 0.05).

Real-time PCR was carried out to evaluate the gene copy numbers of symbionts by comparing the DNA content of symbiont methane monooxygenase and host ACTB and RPL15 genes. Thus the qPCR results could indicate the relative symbiotic bacterial abundance during maintenance. Symbiont quanties from the freshly collected and both maintained mussel groups are shown in Fig. 4. Generally, the three subunits (*pmoA*, *pmoB*, and *pmoC*) displayed similar patterns of relative symbiont abundance. Pearson correlations of the three subunits among individual symbiont quantity were 0.991 to 0.993.

As shown in Fig. 4, symbiont quantity had decreased by 70.5 ∼ 78.4% during the acclimation period, prior to the beginning of the methane treatment experiment, in comparison to the FC mussels. Following this, the symbiont abundance was continually reduced at 7 d and 14 d and was not significantly different between the two groups during this period. By 24 d, the symbiont quantity of the group exposed to methane reached 25.2 ∼ 28.2% of the FC quantity; this level was sustained to 34 d at the conclusion of the experiment. Meanwhile, the symbiont abundance of the group not exposed to methane continued to decline, to under 10% that of the FC group remaining after 34 d. At 24 d and 34 d of laboratory maintenance, all three pmo subunits showed significant differences in symbiont quantity between the two groups.

### Electron microscopy validation

**Figure 5:**
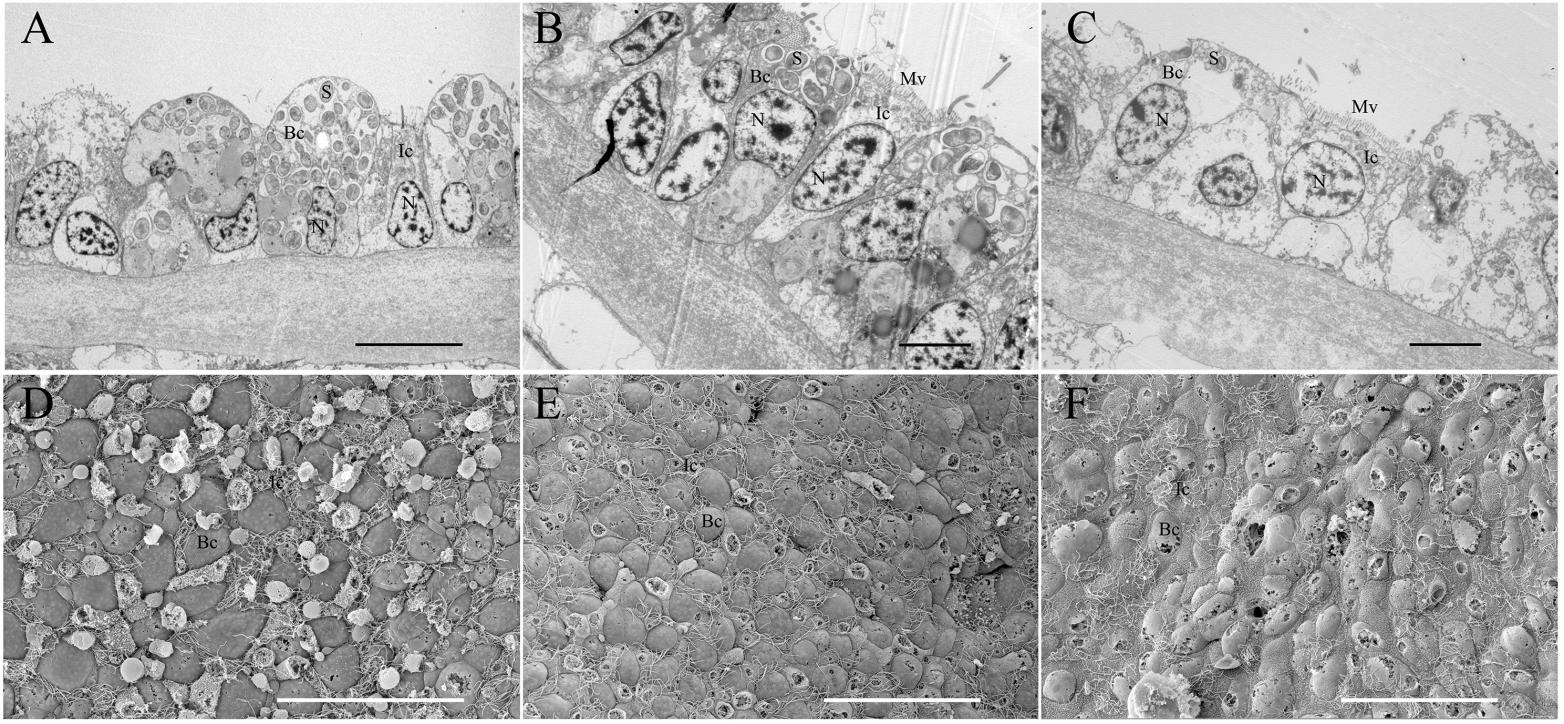
Transmission and scanning electron micrographs of *Bathymodiolus platifrons* gill filaments dissected freshly upon collection (A, D) and after 34 days long-term maintenance under a methane-supplied environment (B, E) or methane-absent environment (C, F). (A–C) cross sections of gills showing symbionts (S) located in bacteriocytes (Bc), intercalary cells (Ic), cell nucleus (N), and microvilli (Mv). Scale bar, (A) 10 µm; (B, C) 5 µm. (D–F) the bacteriocytes (Bc) were smooth and the intercalary cells (Ic) were covered with microvilli. Scale bar: 50 µm.

TEM and SEM were employed to validate the symbiont abundance in the gill bacteriocytes of the FC and 34 d maintained mussels (Fig. 5). TEM revealed that higher densities of coccoid or rod-shaped bacterial symbionts in *B. platifrons* gill bacteriocytes were found in FC mussel (Fig. 5A) than in maintained mussels (Figs. 5B, 5C). The SEM results indicated differences of symbiont abundance under different environments, with cell sizes and contents of bacteriocytes in the FC mussel (Fig. 5D) gills larger than those in the maintained mussels (Figs. 5E, 5F). After 34 days of maintenance, bacteriocytes from both the methane-positive and -negative groups were smaller and flattened, and more so in the methane-negative group (Figs. 5E, 5F). Using ImageJ, we measured the symbiont abundance according to the TEM results. The abundances for FC and maintained mussels were S_Abd_(FC) = 0.190 ± 0.019, S_Abd_(34d, CH_4_ + ) = 0.076 ± 0.007, and *S*_Abd_(34d, CH_4_ − ) = 0.016 ± 0.004. These results confirmed our previous finding that the quantity of symbionts in gill bacteriocytes maintained under methane was higher than that in mussels maintained without methane.

## Discussion

*B. platifrons* owe their high biomass in South China Sea cold seep sites to their ability to adapt and thrive in chemosynthesis-based environments. Previous studies have demonstrated that methanotrophic bacteria located in gill epithelium of *B. platifrons* provide the main carbon source to the host, but the molecular mechanisms of methane oxidization and fixation are still unclear. In this study, we undertook systematic molecular characterization and phylogeny construction of the three subunits of methane monooxygenase (*pmoA*, *pmoB*, and *pmoC*), which is the key enzyme in methane oxidization. Sequence alignment and phylogenetic analysis revealed that the *B. platifrons* endosymbionts shared the greatest sequence similarity and closest phylogenetic distance to known type I methanotrophic bacteria. Endosymbionts in conspecifics located nearby, in similar deep-sea environments, have also been proven to be type I methanotrophs (Fujiwara et al., 2000; Barry et al., 2002). We further compared the 16S rDNA V3∼V4 sequence of *B. platifrons* endosymbionts in the South China Sea (Supplemental File) with that in the Okinawa Trough. BLAST comparison revealed that the similarity between them was 100%. These results further indicate that the endosymbionts of these two conspecifics were probably the same species.

We searched the metagenomic database of the *B. platifrons* using the TBLASTN program and found only one copy for each of the three genes. This result shows that the three genes were all single-copy genes in the *B. platifrons* endosymbiont. However, in the genome of the methanotroph *M. capsulatus* (Bath), two complete copies of *pmoCAB* and a third copy of *pmoC* have been identified (Stolyar et al., 1999; Ward et al., 2004), and in some type II methanotrophs, such as *Methylosinus trichosporium* OB3b and *Methylocystis* sp. strain M, two copies of *pmoC* have been found (Gilbert et al., 2000). The duplicated copies of the *pmo* genes have been found to be functional and contribute to methane oxidization, and different copies of the *pmo* genes could not be discriminated by functional analysis (Stolyar et al., 1999). However, in the *B. platifrons* symbiont, our result demonstrates that a duplication event of the *pmo* genes has not occurred, as has been found in symbionts of *B. azoricus*, *B. heckerae* and *B. brooksi* (Spiridonova et al., 2006; Duperron et al., 2007; Raggi et al., 2013). Consequently, it is reasonable to use the three subunits as specific markers for further phylogenetic or functional analysis in *B. platifrons*.

We also reported the first laboratory maintenance experiment of *B. platifrons*, which allowed for the use of qPCR in detecting changes in the abundance of endosymbiont corresponding to the environmental methane concentration. Previously, FISH was the most popular technique for visualizing and quantifying relative abundances of symbionts, and has been widely applied to studies on bacterial symbiosis in various invertebrates inhabiting deep-sea hydrothermal vents and cold seeps, such as gutless worms, gastropods and shrimp (Blazejak et al., 2005; Suzuki et al., 2005; Suzuki et al., 2006; Tokuda et al., 2008; Fujinoki et al., 2012). According to those studies, FISH was proven to be an indispensable approach in visualizing the distribution or area of symbiont. Relying on image analysis of the fluorescence signal intensity, it was also used to calculate bacterial volumes or relative abundance under different conditions. Nevertheless, the throughput of each FISH treatment was limited, and it was difficult and time-consuming to detect a great many of samples using FISH technique. Because differences existed prevalently between individuals’ symbiont populations in deep-sea invertebrates, this may result in skewed results. In this study, using the qPCR methods, the level of replication achieved in our design helped to avoid misrepresentation caused by individual difference. However, qPCR methods detect the symbiont gene copy numbers, and thus genomes, may respond differently to variations than the whole bacterial volume or area (Duperron et al., 2016). For example, genome amplification without cytokinesis may induce significant increase in symbionts gene copy numbers without corresponding increase in their area or numbers. Therefore, in our work we use electron microscopy to detect the actual symbiont quantity in the bacteriocytes. The results of electron microscopy from the freshly-collected and long-term maintained (34 d) mussels confirmed with the qPCR results. Therefore, real-time qPCR combined with electron microscopy analysis was shown to be an accurate approach for detecting changes in symbiont dynamics in our study.

We found that the symbiont quantity at the beginning of methane treatment experiment was much less than in freshly collected mussels (Fig. 4), with the reduction likely occurring during the one-day acclimation period at atmospheric pressure. A previous study of *B. azoricus* mussels found that cellular stress induced by changes in atmospheric pressure led to increased superoxide dismutase (SOD) activity in the gills of freshly collected mussels, which indicates that decompression may trigger SOD as an antioxidant defense (Martins et al., 2016). SOD plays a central role in protection against oxidative stress in all aerobic organisms, and has been reported to increase in mussel gills exposed to various environmental stresses (Company et al., 2004; Valavanidis et al., 2006). In addition, another stress-related gene *HSP70*, involved in defense and oxidative stress-related mechanisms such as protein folding and cell stress protection molecules, was highly expressed in *B. azoricus* at 24 h after collection (Barros et al., 2015). The overexpression of *HSP70* suggests that severe environmental stress and subsequent antioxidant protective reactions occur during maintenance (Pruski & Dixon, 2007). Taken together, these studies demonstrate high environmental and oxidative stress in freshly collected mussels and continuous perturbation of cellular redox homeostasis during short-term maintenance. Cellular redox homeostasis is necessary for the maintenance of symbioses associations (Mone, Monnin & Kremer, 2014). As oxidative balance requires fine tuning between the symbiont and the host, even a small disturbance in the internal oxidative environment can lead to the breakdown of symbioses (Mone, Monnin & Kremer, 2014). In coral, environmental stressors such as high temperature can induce the increase of ROS (reactive oxygen species) and trigger the cnidarian/dinoflagellate symbiosis breakdown through series of downstream pathways including cell death via apoptosis and necrosis, leading to bleaching (Vidal-Dupiol et al., 2009; Paxton, Davy & Weis, 2013; Weis, 2008). Therefore, acute environmental changes, especially decompression during collection, may cause high cellular oxidative stress and perturbation of cellular redox homeostasis, contributing to the rapid decrease of symbiont abundance seen in the one-day acclimation.

After this initial decline, the relative DNA content of the three *pmo* genes revealed that symbiont abundance gradually declined from 0 d to 7 d and 14 d during short-term maintenance in both group CH_4_ + and CH_4_ − (Fig. 4). The methane concentration at one atmosphere of pressure in the laboratory cannot reach the levels experienced in a deep-sea environment for the group CH_4_ +, and the decreased methane concentration may account for the reduced of symbiont. As measured in this study by gas chromatography, the dissolved methane concentration in aquarium seawater after perfused for 30 min was about 0.338 mM, and the dissolved methane concentration declined over time with methane gas escaping from water. A recent study developed a deep-sea hybrid Raman insertion probe and inserted directly into the chemosynthetic communities near the cold seep at Formosa Ridge in the South China Sea, the same location *B. platifrons* was collected in our study (Zhang et al., in press). Using the *in situ* Raman sensor, the authors detect the fluid inside the chemosynthetic communities. The result indicated that the dissolved CH_4_ concentration in the diffuse fluids under the cold seep chemosynthetic communities was as high as 1.54 mM (Zhang et al., in press). Furthermore, high methane concentrations were also detected in end-member fluids of Izena, Okinawa Trough (7.6 mM) and Sagami Bay (6 mM), where *Bathymodiolus* species based solely on methane oxidation as the energy source distributed (Sakai et al., 1990; Tsunogai et al., 1996; Fujiwara et al., 2000). Thus, the decreased methane concentration of the laboratory environment may the main reason for the declination of symbiont abundance. In further analyses, a close or pressed aquarium can be used for the maintenance of higher *B. platifrons* symbionts abundance.

Significant differences were not detected between CH_4_ + and CH_4_ − groups at 7 d and 14 d after laboratory maintenance. There are two possible reasons for this. First, methylotrophs are known to accumulate intracellular poly-hydroxybutyrate and glycogen/amylopectin as a common carbon storage approach (Hyder, Meyers & Cayer, 1979; Eshinimaev et al., 2002; Rahalkar, Bussmann & Schink, 2007; Puri et al., 2015). Electron microscopy has detected poly-hydroxybutyrate-like inclusions in the gill bacterial cells of *B. platifons* from cold seeps in Sagami Bay, Japan (Barry et al., 2002). This stored carbon can be continuously consumed and provide energy in methylotrophs when methane is removed from the culture (Khadem et al., 2012). Therefore, carbohydrate inclusions can provide energy allowing for the short-term maintenance of *B. platifrons* endosymbionts even without methane supplement. Second, mRNA expression in both groups may show differences. Analysis of the dual symbiotic hydrothermal vent mussel *B. puteoserpentis* revealed that mRNA expression of thiotrophic and methanotrophic symbionts differed between individual mussels, without obvious differences in symbiont relative ratios or distribution (Wendeberg et al., 2012). The regulation of mRNA expression is a rapid response to adapt to short-term environmental variation, such as temporal and spatial gradients of methane at a seep, or reduced sulfur compounds and oxygen. Resulting changes in symbiont abundance may take much longer than the modification of gene expression, and may occur only in response to long-term environmental changes (Kádár et al., 2005; Riou et al., 2008). Measurements of the mRNA expression of the methane oxidization genes should be combined with methanotrophic symbiont abundance to better understand methane metabolism in *B. platifrons* in future studies.

At longer maintenance time-points (24 d and 34 d), the symbiont quantities observed in mussels exposed to supplementary methane likely reached a stable level as the bacteria became acclimatized to a lower CH_4_ concentration and reached a new equilibrium. In contrast, symbiont abundance significantly declined in the absence of methane in the other group, likely due to the lack of an energy source. This phenomenon has been reported in other deep-sea bivalves as well. Sulfur-oxidizing symbiotic bacteria in the deep-sea clam *Calyptogena okutanii* significantly decrease after 57 days maintenance without sulfur supply, and disappear completely after 91 days (Ohishi et al., 2016). Endosymbionts also gradually disappear from *B. azoricus* bacteriocytes in animals kept in seawater free of dissolved sulfide for up to 30 days (Kádár et al., 2005). These works demonstrate that the maintenance of endosymbionts relies on a continuous supply of substrate, and a long-term absence of an energy source causes considerable symbiont loss in deep-sea bivalves.

## Conclusion

The deep-sea mussel *B. platifrons* occupies an overwhelmingly dominant position in the cold seep at the South China Sea because of its successful association with methanotrophic bacteria, which confers a nutritional advantage by providing energy from methane oxidization. In the present study, we report the identification of key methane-oxidizing genes encoding methane monooxygenase (*pmoA*, *pmoB*, and *pmoC*). Furthermore, using sustained laboratory maintenance, we analyzed the influence of environmental methane on symbiont abundance. Dynamic changes in symbiont abundance were evaluated by calculating the relative DNA content of methane monooxygenase using real-time PCR. This study enriches our knowledge of the molecular basis of a typical methanotrophic endosymbiosis in *B. platifrons*, and provides a feasible model system for further analysis of methane oxidization and carbon fixation in deep-sea mussels.

##  Supplemental Information

10.7717/peerj.3565/supp-1 Supplemental Information 1 qPCR raw data of pomA, pmoB and pmoC The qPCR raw data of symbiont pomA, pmoB and pmoC genes and host ACTB and RPL15.

10.7717/peerj.3565/supp-2 Table S1 Similarity analysis methane monooxygenase between *Bathymodiolus platifrons* endosymbiont and other methanotroph species Methanotroph species, phylogenetic group and accession number of methane monooxygenase and the percentage identities with *Bathymodiolus platifrons* endosymbiont pmoA, pmoB and pmoC amino acids.

10.7717/peerj.3565/supp-3 Fig. S1 Nucleotide and deduced amino acid sequence of pmoA from *Bathymodiolus platifrons* “*” indicates the stop codon.

10.7717/peerj.3565/supp-4 Fig. S2 Nucleotide and deduced amino acid sequence of pmoB from *Bathymodiolus platifrons* “*” indicates the stop codon.

10.7717/peerj.3565/supp-5 Fig. S3 Nucleotide and deduced amino acid sequence of pmoC from *Bathymodiolus platifrons* “*” indicates the stop codon.

10.7717/peerj.3565/supp-6 Fig. S4 Phylogenetic reconstruction of methane monooxygenase based on Bayesian analysis of concatenated coding sequences The posterior probability is indicated at the tree nodes. Methane monooxygenase of the *Bathymodious platifrons* symbiont is marked in bold. Bar: 0.3 substitutions per nucleotide position.

10.7717/peerj.3565/supp-7 Fig. S5 Phylogenetic reconstruction of methane monooxygenase subunits pmoA, pmoB, and pmoC based on Bayesian analysis of protein sequences The posterior probability is indicated at the tree nodes. PmoA, pmoB, and pmoC of the *Bathymodious platifrons* symbiont are marked in bold. Bar: 0.4 substitutions per amino acid site.

10.7717/peerj.3565/supp-8 Fig. S6 Phylogenetic reconstruction of methane monooxygenase subunits pmoA, pmoB, and pmoC based on Bayesian analysis of coding sequences The posterior probability is indicated at the tree nodes. PmoA, pmoB, and pmoC of the *Bathymodious platifrons* symbiont are marked in bold. Bar: 0.5 substitutions per nucleotide position.

10.7717/peerj.3565/supp-9 Supplemental Information 2 Coding sequences of pmo genes

10.7717/peerj.3565/supp-10 Supplemental Information 3 Protein sequences of pmo genes

10.7717/peerj.3565/supp-11 Supplemental Information 4 16s V3-V4 rDNA sequence of *Bathymodiolous platifrons* endosymbiont

## Additional Information and Declarations

### DNA Deposition

The following information was supplied regarding the deposition of DNA sequences: The methane monooxygenase subunits (pmoA, pmoB and pmoC) sequences described here are accessible via GenBank accession numbers KY393122, KY393123, and KY393124 and are also available in the Supplemental Information.

### Data Availability

The following information was supplied regarding data availability: The raw data has been supplied as a Supplementary File.
