# Supplementary material for: Molecular identification of methane monooxygenase and quantitative analysis of methanotrophic endosymbionts under laboratory maintenance in Bathymodiolus platifrons from the South China Sea
Source: PeerJ. 2017 Aug 7;5:e3565. doi: 10.7717/peerj.3565 (PMC5553348; doi:10.7717/peerj.3565)
Supplement: Table S1 — Methanotroph species, phylogenetic group and accession number of methane monooxygenase and the percentage identities with Bathymodiolus platifrons endosymbiont pmoA, pmoB and pmoC amino acids. [file peerj-05-3565-s002.docx]

**Table S1** Methanotroph species, phylogenetic group and accession numbers of methane monooxygenase used in the sequence similarity and phylogenetic analysis, and the percentage identities with *Bathymodiolus platifrons* endosymbiont pmoA, pmoB and pmoC amino acids.

| **Protein** | **Methanotroph species** | **Phylogenetic group** | **Accession number** | ***Bathymodiolus* *platifrons* endosymbiont** | | |
| --- | --- | --- | --- | --- | --- | --- |
|  |  |  |  | **pmoA I%** | **pmoB I%** | **pmoC I%** |
| pmoA | *Methylomonas methanica* | Type I | WP_013817026 | 93.7 | 16.1 | 46 |
|  | *Methylomonas koyamae* | Type I | WP_064041206 | 92.7 | 16.1 | 45.6 |
|  | *Methylobacter luteus* | Type I | WP_027159170 | 92.9 | 17.5 | 46.4 |
|  | *Methylobacter whittenburyi* | Type I | WP_036297036 | 92.7 | 18.1 | 46.8 |
|  | *Methylomicrobium buryatense* | Type I | WP_017841993 | 91.1 | 17.1 | 46.2 |
|  | *Methylomicrobium alcaliphilum* 20Z | Type I | CCE22213 | 90.9 | 17.3 | 46.6 |
|  | *Methylococcus capsulatus* (Bath) | Type X | AAB49821 | 87.3 | 16.5 | 47.4 |
|  | *Methylocaldum szegediense* | Type X | WP_026609851 | 85.5 | 16.1 | 46.6 |
|  | *Methylocaldum* sp. T025 | Type X | BAF49650 | 85.5 | 16.1 | 46.6 |
|  | *Methylocaldum* sp. 14B | Type X | WP_077732836 | 85.3 | 16.3 | 46.6 |
|  | *Methylocystis parvus* | Type II | WP_016921576 | 77.8 | 16.9 | 45.6 |
|  | *Methylosinus* sp. LW4 | Type II | WP_018265987 | 78.2 | 17.5 | 45.4 |
|  | *Methylocystis* sp. SC2 | Type II | CAE48352 | 77.2 | 16.9 | 45.8 |
| pmoB | *Methylomonas methanica* | Type I | WP_013817027 | 16.7 | 86.3 | 11.7 |
|  | *Methylomonas koyamae* | Type I | WP_064041205 | 16.9 | 85.3 | 11.3 |
|  | *Methylobacter luteus* | Type I | WP_027159171 | 16.7 | 87.1 | 12.1 |
|  | *Methylobacter whittenburyi* | Type I | WP_036297856 | 16.7 | 88.5 | 12.3 |
|  | *Methylomicrobium buryatense* | Type I | WP_017841994 | 15.9 | 84.5 | 12.3 |
|  | *Methylomicrobium alcaliphilum* 20Z | Type I | CCE22214 | 16.1 | 84.7 | 11.7 |
|  | *Methylococcus capsulatus* (Bath) | Type X | AAB49822 | 15.1 | 75.4 | 11.9 |
|  | *Methylocaldum szegediense* | Type X | WP_026609851 | 15.3 | 74.4 | 11.3 |
|  | *Methylocaldum* sp. T025 | Type X | BAF49661 | 14.5 | 71.2 | 10.5 |
|  | *Methylocaldum* sp. 14B | Type X | WP_077732837 | 24.4 | 66.9 | 21.2 |
|  | *Methylocystis parvus* | Type II | WP_016921577 | 14.3 | 52.2 | 8.9 |
|  | *Methylosinus* sp. LW4 | Type II | WP_018265986 | 13.9 | 52.4 | 7.9 |
|  | *Methylocystis* sp. SC2 | Type II | CAE48353 | 13.9 | 52.6 | 7.7 |
| pmoC | *Methylomonas methanica* | Type I | WP_013817025 | 46.8 | 11.3 | 93.8 |
|  | *Methylomonas koyamae* | Type I | WP_064041207 | 46.4 | 11.5 | 93.7 |
|  | *Methylobacter luteus* | Type I | WP_027159169 | 46.4 | 11.5 | 93.1 |
|  | *Methylobacter whittenburyi* | Type I | WP_036297034 | 46.2 | 11.1 | 93.8 |
|  | *Methylomicrobium buryatense* | Type I | WP_017841992 | 46.2 | 11.9 | 93.1 |
|  | *Methylomicrobium alcaliphilum* 20Z | Type I | CCE22212 | 46.2 | 11.5 | 92.9 |
|  | *Methylococcus capsulatus* (Bath) | Type X | AAB49820 | 44.2 | 12.5 | 84.7 |
|  | *Methylocaldum szegediense* | Type X | WP_026609851 | 43.8 | 10.9 | 83.5 |
|  | *Methylocaldum* sp. T025 | Type X | BAF49659 | 43.8 | 10.9 | 83.5 |
|  | *Methylocaldum* sp. 14B | Type X | WP_077732835 | 44 | 11.3 | 83.9 |
|  | *Methylocystis parvus* | Type II | WP_016921575 | 46.4 | 12.1 | 77 |
|  | *Methylosinus* sp. LW4 | Type II | WP_018265988 | 45.8 | 11.9 | 76.4 |
|  | *Methylocystis* sp. SC2 | Type II | CAE48351 | 45.6 | 12.3 | 77.2 |

I%: percentage identity, calculated by clustal W methods in MegAlign software of DNAstar.
