# Supplementary figures and images for: Molecular identification of methane monooxygenase and quantitative analysis of methanotrophic endosymbionts under laboratory maintenance in Bathymodiolus platifrons from the South China Sea
Source: PeerJ. 2017 Aug 7;5:e3565. doi: 10.7717/peerj.3565 (PMC5553348; doi:10.7717/peerj.3565)

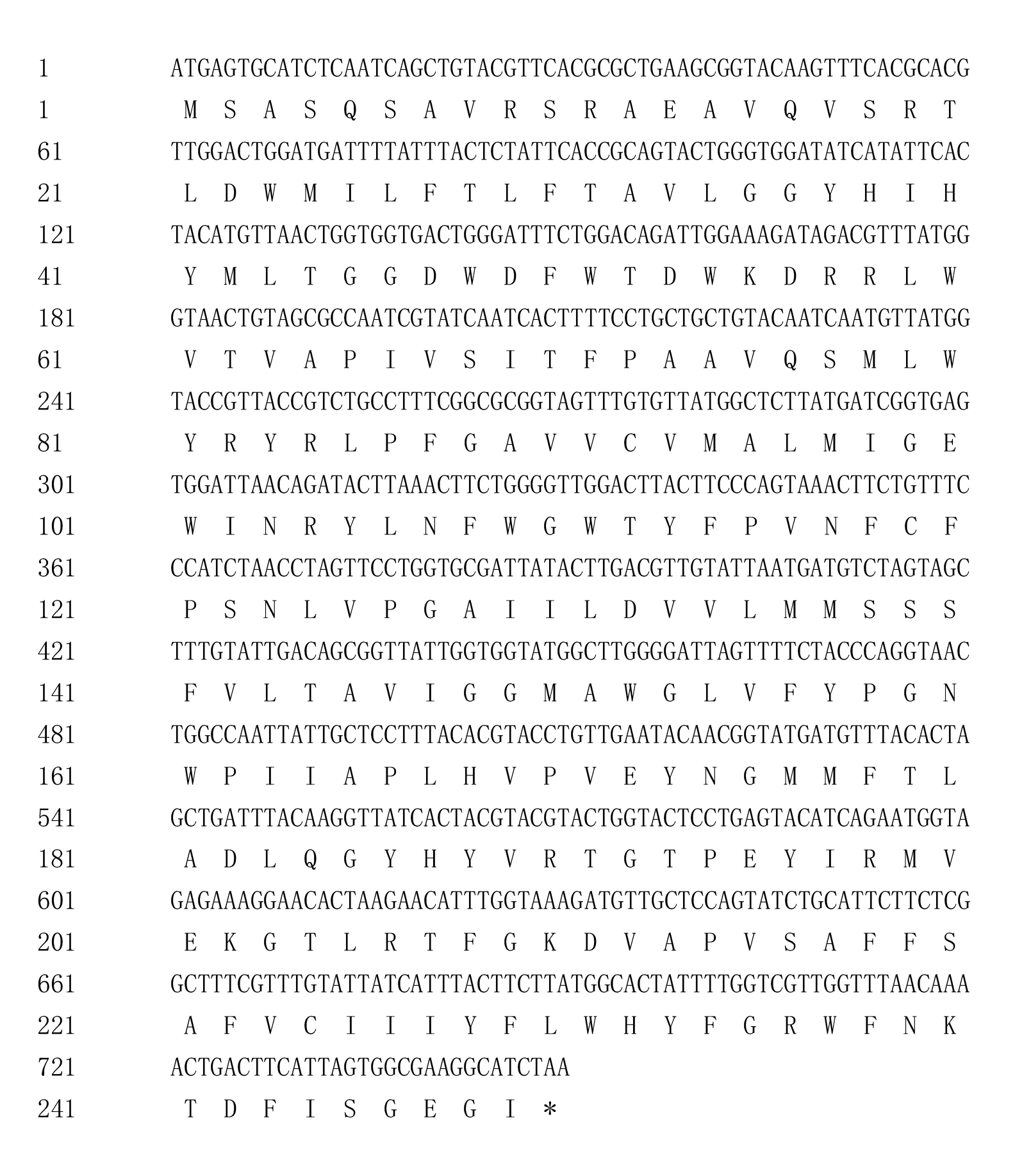

Supplement: Fig. S1 — “*” indicates the stop codon. [file peerj-05-3565-s003.png]

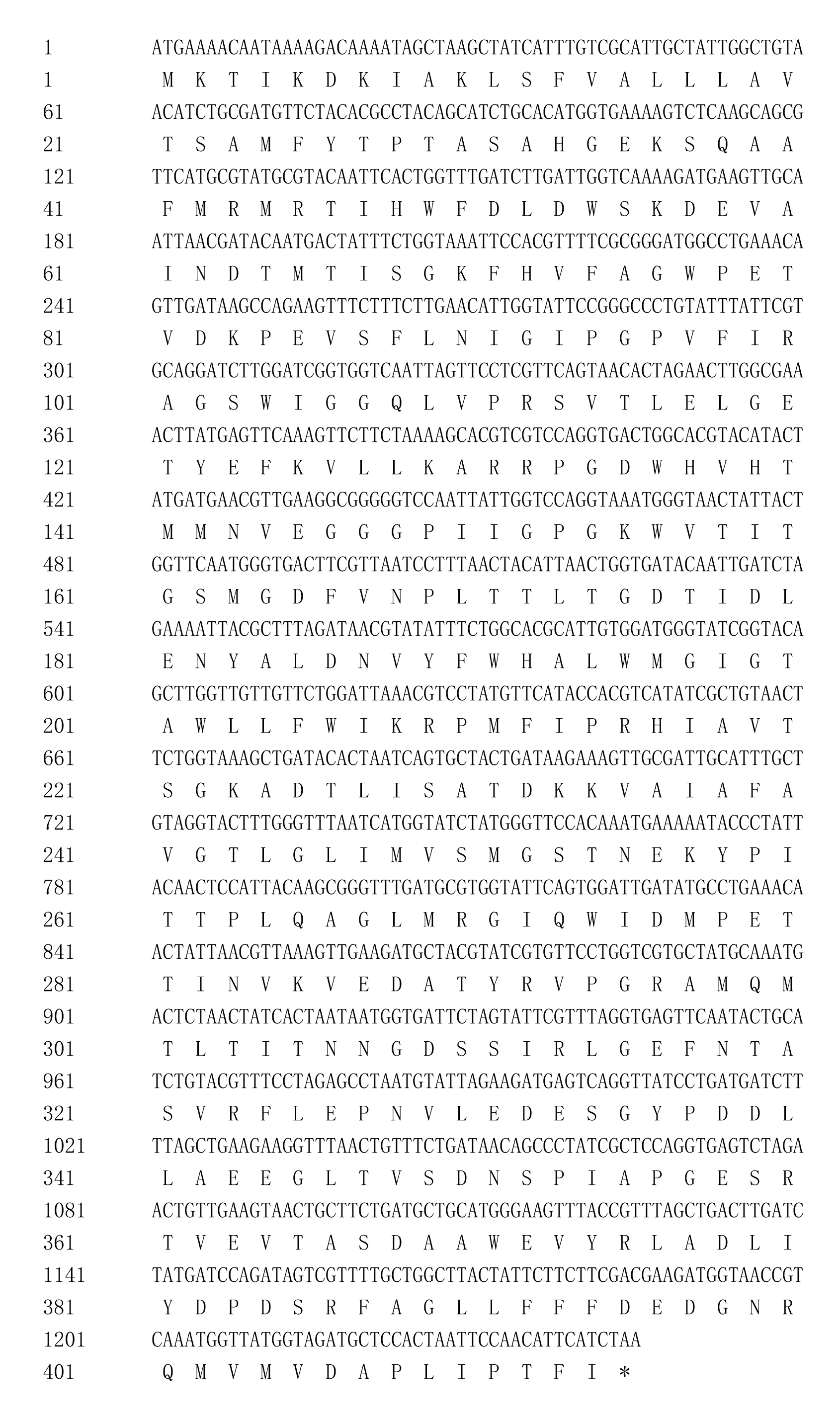

Supplement: Fig. S2 — “*” indicates the stop codon. [file peerj-05-3565-s004.png]

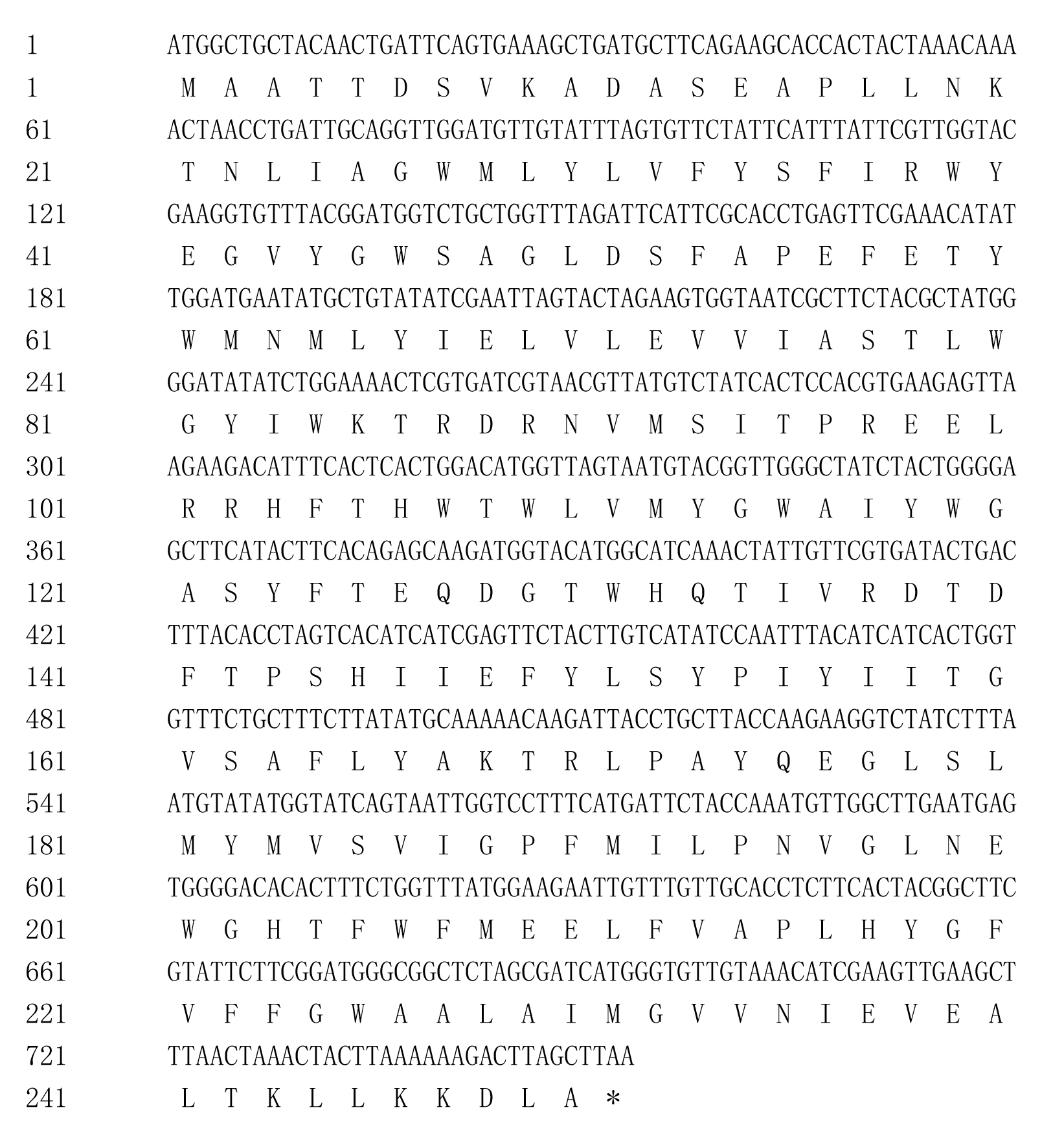

Supplement: Fig. S3 — “*” indicates the stop codon. [file peerj-05-3565-s005.png]

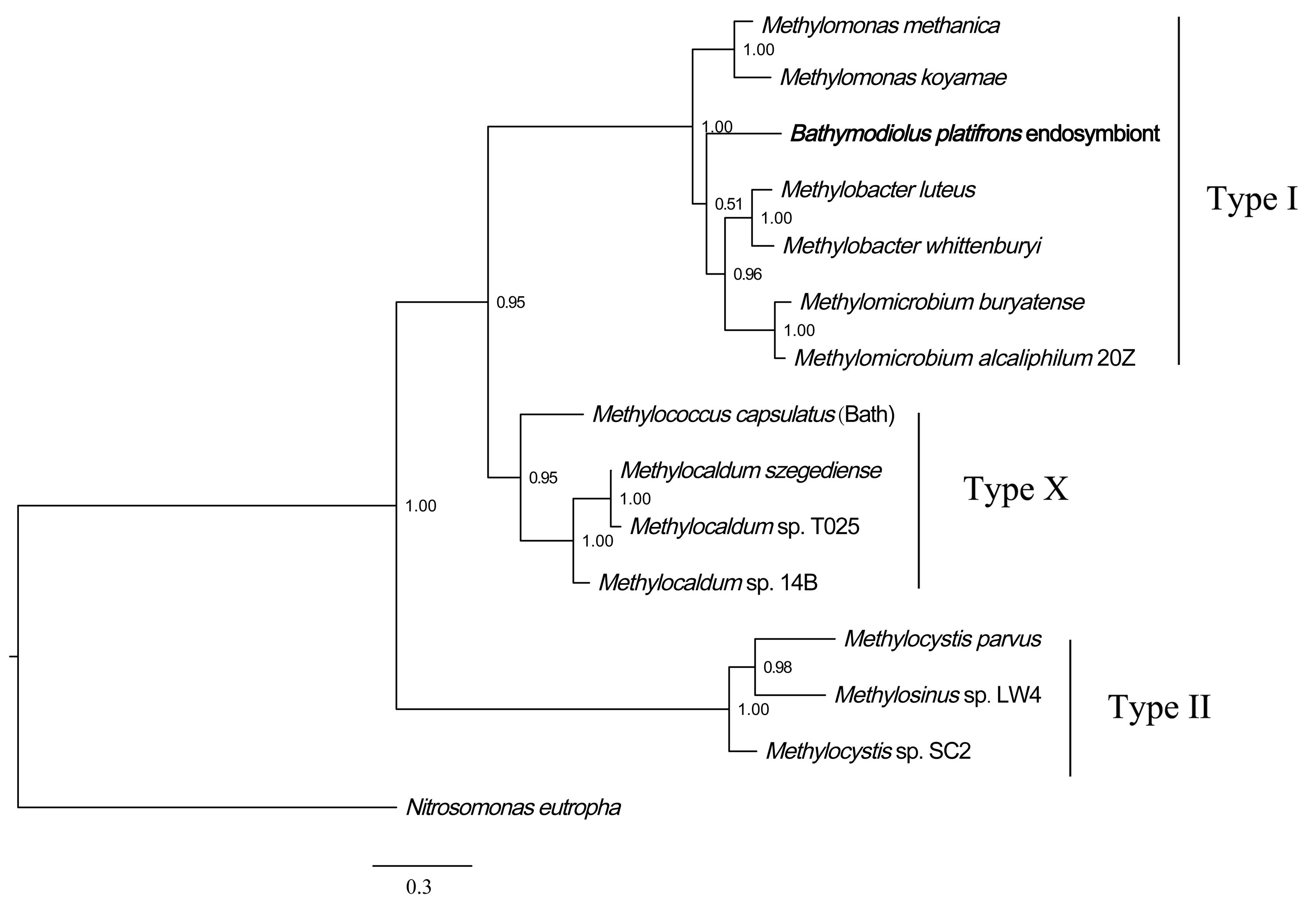

Supplement: Fig. S4 — The posterior probability is indicated at the tree nodes. Methane monooxygenase of the Bathymodious platifrons symbiont is marked in bold. Bar: 0.3 substitutions per nucleotide position. [file peerj-05-3565-s006.png]

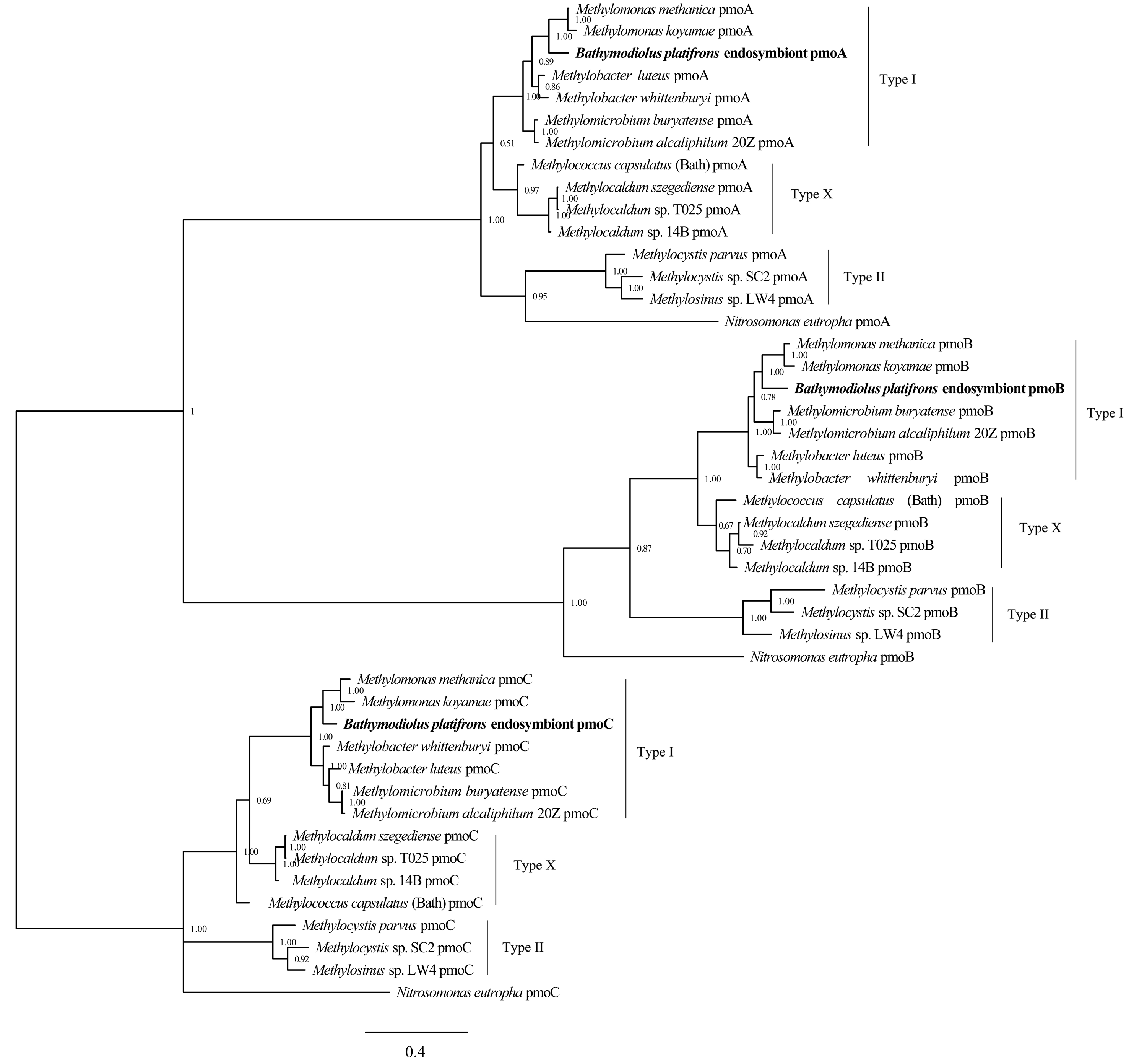

Supplement: Fig. S5 — The posterior probability is indicated at the tree nodes. PmoA, pmoB, and pmoC of the Bathymodious platifrons symbiont are marked in bold. Bar: 0.4 substitutions per amino acid site. [file peerj-05-3565-s007.png]

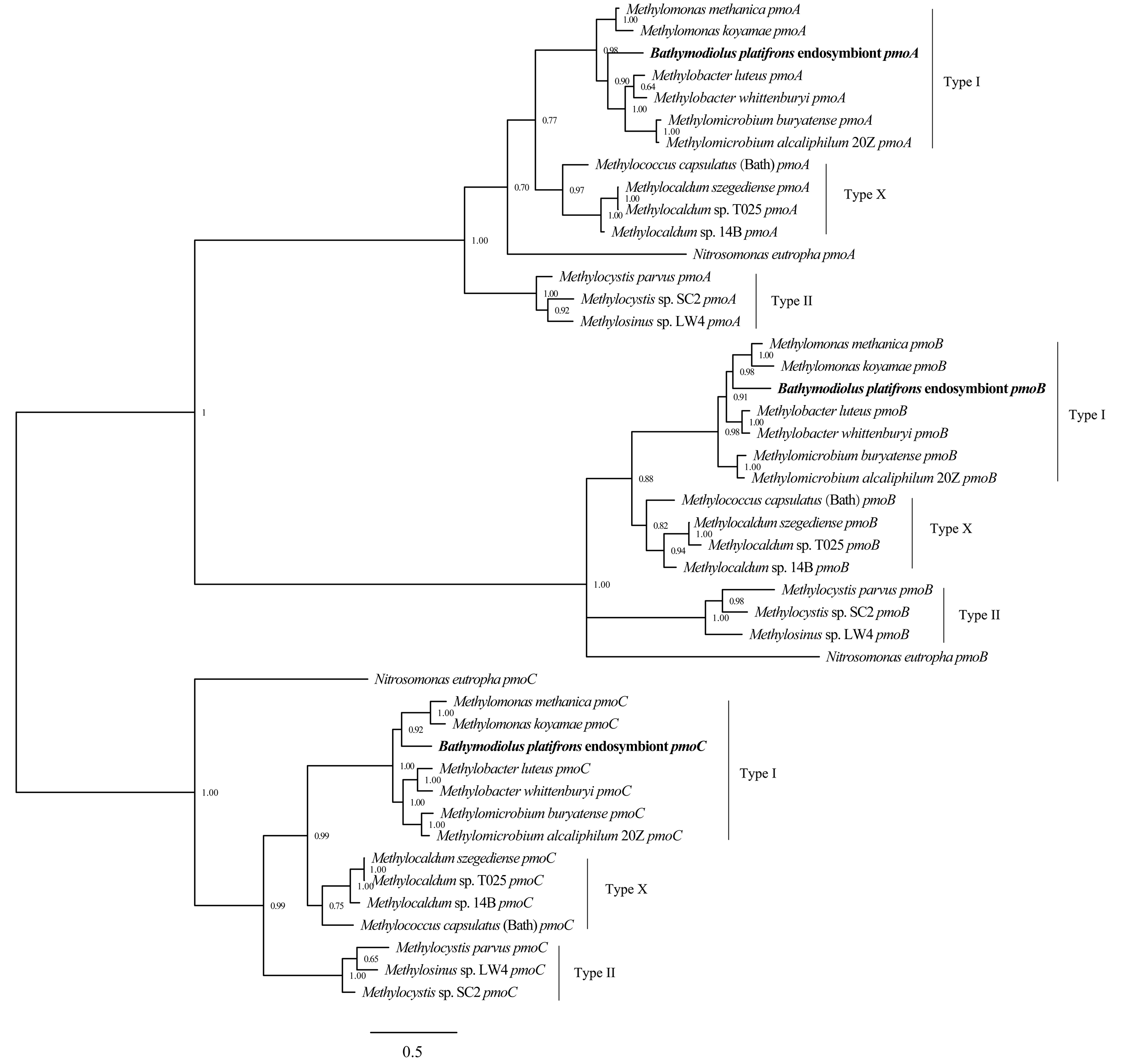

Supplement: Fig. S6 — The posterior probability is indicated at the tree nodes. PmoA, pmoB, and pmoC of the Bathymodious platifrons symbiont are marked in bold. Bar: 0.5 substitutions per nucleotide position. [file peerj-05-3565-s008.png]
